# Transcriptomic and Proteomic Responses of Sweetpotato Whitefly, *Bemisia tabaci*, to Thiamethoxam

**DOI:** 10.1371/journal.pone.0061820

**Published:** 2013-05-09

**Authors:** Nina Yang, Wen Xie, Xin Yang, Shaoli Wang, Qingjun Wu, Rumei Li, Huipeng Pan, Baiming Liu, Xiaobin Shi, Yong Fang, Baoyun Xu, Xuguo Zhou, Youjun Zhang

**Affiliations:** 1 Department of Plant Protection, Institute of Vegetables and Flowers, Chinese Academy of Agricultural Sciences, Beijing, P. R. China; 2 Department of Entomology, University of Kentucky, Lexington, Kentucky, United States of America; Children's Medical Research Institute, Australia

## Abstract

**Background:**

The sweetpotato whitefly, *Bemisia tabaci* (Hemiptera: Aleyrodidae), is one of the most widely distributed agricultural pests. Although it has developed resistance to many registered insecticides including the neonicotinoid insecticide thiamethoxam, the mechanisms that regulate the resistance are poorly understood. To understand the molecular basis of thiamethoxam resistance, “omics” analyses were carried out to examine differences between resistant and susceptible *B. tabaci* at both transcriptional and translational levels.

**Results:**

A total of 1,338 mRNAs and 52 proteins were differentially expressed between resistant and susceptible *B. tabaci*. Among them, 11 transcripts had concurrent transcription and translation profiles. KEGG analysis mapped 318 and 35 differentially expressed genes and proteins, respectively, to 160 and 59 pathways (p<0.05). Thiamethoxam treatment activated metabolic pathways (e.g., drug metabolism), in which 118 transcripts were putatively linked to insecticide resistance, including up-regulated glutathione-S-transferase, UDP glucuronosyltransferase, glucosyl/glucuronosyl transferase, and cytochrome P450. Gene Ontology analysis placed these genes and proteins into protein complex, metabolic process, cellular process, signaling, and response to stimulus categories. Quantitative real-time PCR analysis validated “omics” response, and suggested a highly overexpressed P450, *CYP6CX1*, as a candidate molecular basis for the mechanistic study of thiamethoxam resistance in whiteflies. Finally, enzymatic activity assays showed elevated detoxification activities in the resistant *B. tabaci*.

**Conclusions:**

This study demonstrates the applicability of high-throughput omics tools for identifying molecular candidates related to thiamethoxam resistance in an agricultural important insect pest. In addition, transcriptomic and proteomic analyses provide a solid foundation for future functional investigations into the complex molecular mechanisms governing the neonicotinoid resistance in whiteflies.

## Background

Neonicotinoids, a relatively new class of synthetic insecticides, have been used primarily to control aphids, leafhoppers, whiteflies, and other sap-sucking pests [1], [2]. Imidacloprid and thiamethoxam, two major neonicotinoids on current market, specifically act on the insect's nicotinic acetylcholine receptors (nAChR) within the central nervous system [2], [3]. Extensive and repetitive use of neonicotinoids have led to the development of resistance in the fruit fly *Drosophila melanogaster*, the green peach aphid *Myzus persicae*, the house fly *Musca domestica L*, the brown planthopper *Nilaparvata lugens* [4], [5], [6], [7], and the sweetpotato whitefly *Bemisia tabaci* [8], [9].

*Bemisia tabaci* is an important pest of arable and horticultural crops in the temperate regions of the world. It has a broad host range and can cause tremendous damages directly by feeding and indirectly by transmitting 115 species of begomoviruses [10], [11]. *Bemisia tabaci* complex contains more than 24 morphologically indistinguishable biotypes, although recent studies have suggested these are genetically distinct cryptic species [12], [13], [14]. During the past two decades, *B. tabaci* biotype B, one of the most invasive and destructive species/biotypes, has been introduced into at least 54 countries from its origin in the Middle East-Asia Minor region and become a world-wide invasive whitefly species [12], [13]. In China, *B. tabaci* was first recorded in the late 1940s [15]. However, the crop damages and economic losses caused by this phloem-feeding insect had not been severe until the introduction of *B. tabaci* B biotype in the mid-1990s [16]. Since then, B biotype has quickly displaced the indigenous *B. tabaci* populations, rapidly invaded the entire country, and has led to serious yield losses in many crops [17].

The management of *B. tabaci* has been relied heavily on synthetic insecticides. As a result, pesticide resistance has been developed in *B. tabaci* in many parts of the world. In Israel and Spain, for example, *B. tabaci* field populations were found highly resistant to thiamethoxam [8], [18], [19]. In Crete, *B. tabaci* developed over 1,000-fold resistance to imidacloprid in comparison to its susceptible counterpart in the field [20]. In China, field collected *B. tabaci* has developed high level of resistance to both imidacloprid and thiamethoxam [21].

Study of insecticide resistance relies heavily on detailed biochemical, genetic, and molecular analyses. In general, the development of insecticide resistance involves one of the following mechanisms: 1) the over-expression of enzymes that break down or bind to (sequester) the pesticide; 2) target-site modifications (mutations) that reduce sensitivity to the insecticide; or 3) reduced penetration of the pesticide through the insect cuticle [7], [22], [23]. For example, a point mutation (Y151S) in two nAChR subunits led to the development of neonicotinoid resistance in *N. lugens* [7]. Other studies have suggested that the resistance to neonicotinoid pesticides was associated with over-expression of detoxification enzyme cytochrome P450 *CYP6G1* in *D. melanogaster* [4]. Similarly, resistance to neonicotinoid insecticides in the green peach aphid *M. persicae* has been linked with the over-expression of *CYP6CY3* [5].

In *B. tabaci*, neonicotinoid resistance has yet to be correlated with target-site modification, but has been associated with elevated expression of specific genes. For example, over-expression of a cytochrome P450, *CYP6CM1*, has been linked to imidacloprid resistance in *B. tabaci* [24]. In addition, *B. tabaci* resistance to neonicotinoids, especially to thiamethoxam, has been associated with elevated activities of detoxification enzymes [25], [26]. Most recently, the molecular basis of thiamethoxam resistance in *B. tabaci* was investigated using the suppression subtractive hybridization (SSH) cDNA library approach [27]. Based on the results of the differential screening, 298 and 209 clones were picked and sequenced, respectively, from the forward and reverse cDNA libraries, representing up- and down-regulated genes between the thiamethoxam-resistant and -susceptible *B. tabaci*. BLASTX analysis matched 24.5% of these differentially expressed transcripts to genes with known functions. Among them, a NAD-dependent methanol dehydrogenase-like EST from *B.tabaci* was substantially overexpressed in the resistant whiteflies (∼12-fold). Despite recent progresses, molecular mechanisms underlying *B. tabaci* resistance to neonicotinoids remain poorly understood.

Functional genomics and proteomics provide an unprecedented opportunity for scientific community to gain a global understanding of the spatial and temporal dynamics of molecular and cellular processes in a living organism and notably facilitate the analysis of genetics and metabolic pathways governing these processes [28], [29]. Also, the application of genomic tools to previously intractable cases of insecticide resistance should greatly expand our understanding of how insecticide resistance evolves and can be avoided or managed [30], [31]. In this study, we compared thiamethoxam resistant and susceptible *B. tabaci* at transcriptome (RNA-seq) and proteome (iTRAQ) level to enhance our genetic and molecular understanding of the thiamethoxam resistance in an agriculturally important insect pest.

## Results

### Omics analyses

Thiamethoxam susceptible and resistant *B. tabaci* transcriptomes were sequenced individually, generating approximately 12 million raw reads for each library (Table 1). After removal of the low quality reads, the total number of clean reads per library ranged from 11–12 million. To reveal the molecular events underlying transcriptomic profiles, sequence reads were mapped to a reference transcriptome containing both B and Q biotypes [32], [33]. Among the two RNA-seq libraries, 77.69% and 79.03% of reads were mapped to a gene in the reference database with a perfect match ratio of 64.55% and 65.45%, respectively (Table 1). The percentage of reads mapped to unique genes were approximately 40%; representing a critical subset of RNA-seq libraries.

**Table 1:** Summary of RNA-seq metrics from *B. tabaci* transcriptomes.

Metric	TH-S	TH-2000
Total reads	12,350,974	11,667,849
Total base-pair (bp)	605,197,726	571,724,601
Mapped reads (%)	9,761,475 (79.03%)	9,065,171 (77.69%)
Perfect match (%)^1^	8,083,349 (65.45%)	7,531,325 (64.55%)
Unique match (%)^2^	4,980,239 (40.32%)	4,769,926 (40.88%)

1 The percentage of reads that mapping to reference perfectly.

2 The percentage of reads that unambiguous mapped to reference.

The total number of mass spectrometry detected in *B. tabaci* proteomes was 39316, representing 5765 peptide spectra and 2226 distinct peptides (Table 2, Dataset S1). Of the 1005 peptides identified, more than 70% (711) were assigned to a putative protein by homology search against non-redundant (NR) database; leaving approximately 30% (297) of the peptides unidentified. Among these annotated proteins, 372 were either hypothetical, putative, or predicted.

**Table 2:** Summary of iTRAQ metrics from *B. tabaci* proteomes.

Metrics	Number
Total spectra	39316
Unique spectra	2226
Matched protein	1005
Differentially expressed protein	52

### Differentially expressed genes between susceptible and resistant *B. tabaci*

**Figure 1:**
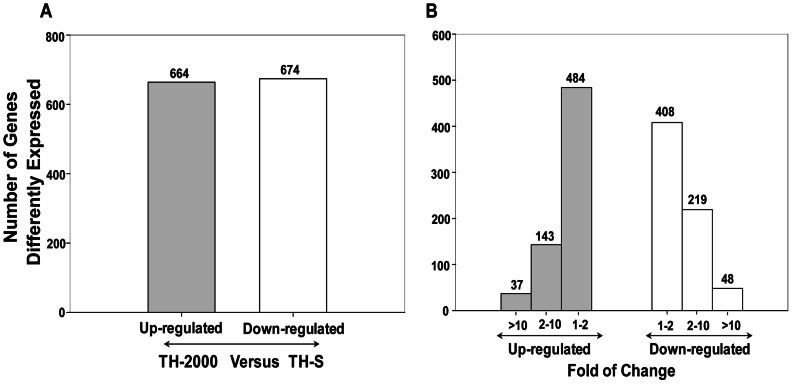
Differentially expressed genes between thiamethoxam resistance and susceptible *B. tabaci*. (A) All 338 differentially expressed genes between the two *B. tabaci* strains were selected with a cutoff value of FDR≤0.001 and |log 2 Ratio|≥1. (B) The distribution of differentially expressed genes based on fold of changes.

Following thiamethoxam treatment, a total of 664 and 674 up- and down-regulated transcripts, respectively, were differentially expressed (FDR≤0.001 and |log2Ratio|≥1) between susceptible (TH-S) and resistant (TH-2000) whiteflies (Figure 1A). Majority of these transcripts (400, ∼66%), however, expressed within 1–2 fold differences (Figure 1B).

**Figure 2:**
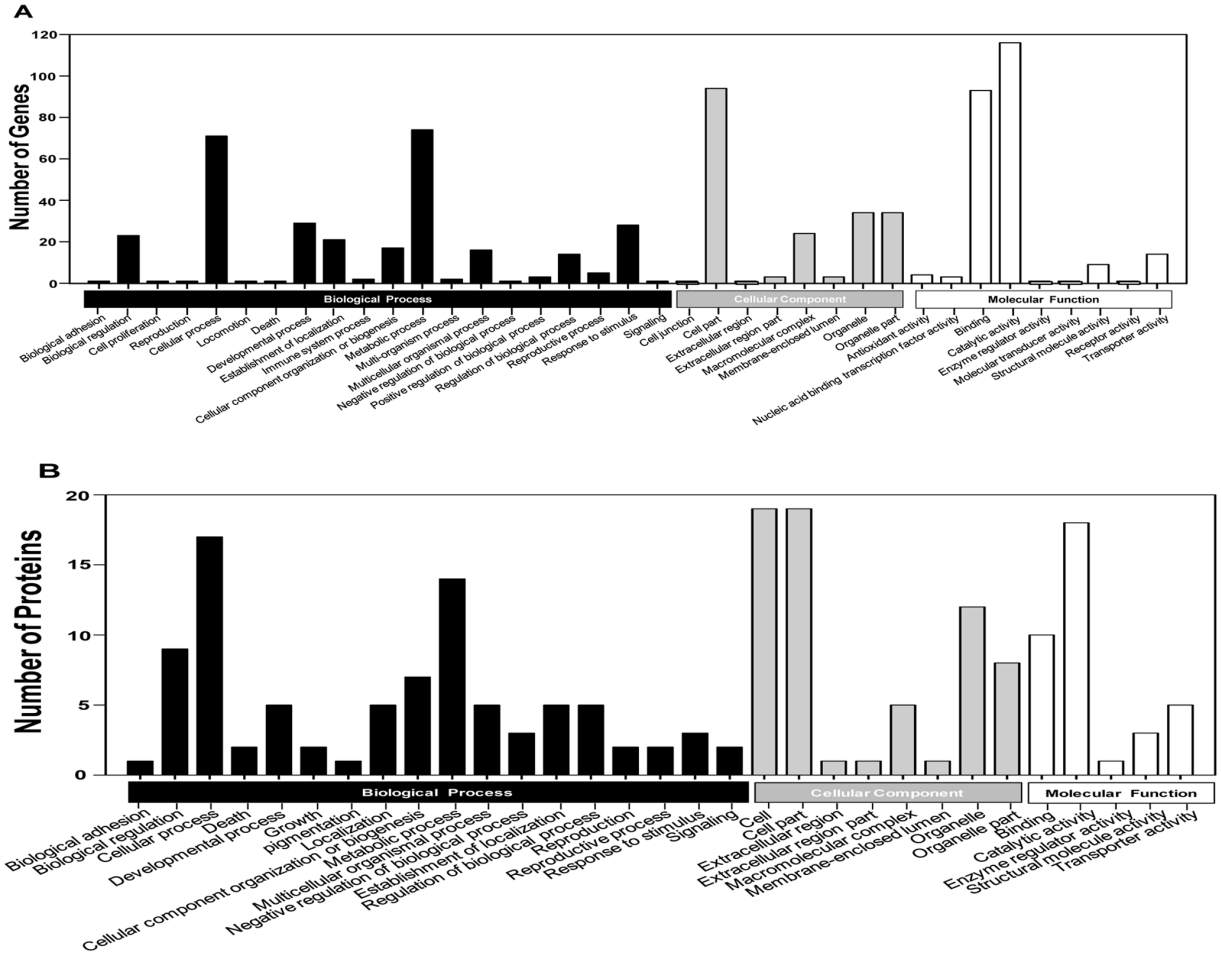
Gene Ontology classification of differentially expressed genes and proteins between thiamethoxam resistance and susceptible ***B.*** **tabaci**** **.** **** The differentially expressed genes or proteins are grouped into three hierarchically-structured GO terms, biological process, cellular component, and molecular function. The y-axis indicates the number of genes or proteins in each GO term. (A) Differentially expressed genes identified by RNA-seq. (B) Differentially expressed proteins identified by iTRAQ.

Table S1 showed the GO classification of 1338 differentially expressed transcripts between TH-S and TH-2000 whiteflies (2∼fold change, FDR≤0.001). Using Blast2GO, 186 differentially expressed transcripts were able to assign to 37 GO classes (Figure 2A). Majority of these genes were assigned to metabolic process, cellular process, multi-organismal process, response to stimulus, binding, catalytic, organelle, cell, and cell part (Table S1). In the up- regulated group, most of genes were assigned to the cathepsin b-like proteinase, NADH-dehydrogenase, glutathione-S-transferase (GST) genes. To investigate their biological functions, 318 differentially expressed genes were mapped to 160 pathways in the KEGG database. Among them, 40 pathways were substantially enriched (p-value≤0.05), such as “Metabolic pathway” and “Drug metabolism pathway” (Table 3). Specifically, 21 genes encoding enzymes in drug metabolism pathway were highly enriched, including 4 cytochrome P450s and 5 GSTs (Table S2). Interestingly, we also found 53 up-regulated and 65 down-regulated genes in metabolic pathways (Table S3). Up-regulated transcripts included GST, cytochrome P450, glucosyl/glucuronosyl transferases, udp glucuronosyl transferases, nadh dehydrogenase, arginine kinase and cytochrome c oxidase, whereas down-regulated ones were 1-acyl-sn-glycerol-3-phosphate acyltransferase, hemocyanin subunit, NADH- dehydrogenase, aconitase and cytochrome p450. It is worth noting that some of these differentially expressed genes were linked to central nervous system (CNS) diseases pathways, such as Parkinson's disease and Alzheimer's disease. A plausible explanation is that nAChRs, the target site for neonicotinoid insecticides, reside in the CNS.

**Table 3:** Significantly enriched KEGG pathways in *B. tabaci* transcriptome.

Pathway	Gene		P-value	Pathway ID
	Dif Expressed^1^	Expressed^2^		
Starch and sucrose metabolism	34	721	4.97E-14	ko00500
Antigen processing and presentation	25	400	2.90E-13	ko04612
Metabolic pathways	118	6788	2.08E-11	ko01100
Galactose metabolism	21	447	4.32E-09	ko00052
Drug metabolism - cytochrome P450	21	553	1.63E-07	ko00982
Metabolism of xenobiotics by cytochrome P450	20	543	5.19E-07	ko00980
Lysosome	25	893	3.05E-06	ko04142
Ascorbate and aldarate metabolism	13	273	3.46E-06	ko00053
Viral myocarditis	13	294	7.72E-06	ko05416
Methane metabolism	6	59	2.45E-05	ko00680
Pentose and glucuronate interconversions	13	330	2.61E-05	ko00040
Steroid hormone biosynthesis	16	518	6.08E-05	ko00140
Retinol metabolism	16	567	0.000171754	ko00830
Amyotrophic lateral sclerosis (ALS)	9	203	0.000190324	ko05014
Porphyrin and chlorophyll metabolism	11	336	0.000527308	ko00860
Parkinson's disease	16	631	0.000555731	ko05012
Oxidative phosphorylation	15	571	0.000573791	ko00190
Pyruvate metabolism	11	354	0.000809227	ko00620
Citrate cycle (TCA cycle)	9	256	0.001017943	ko00020
Phototransduction	5	85	0.001512066	ko04744
Olfactory transduction	6	127	0.001616717	ko04740
Fatty acid biosynthesis	7	173	0.0016526	ko00061
Tight junction	14	577	0.001826685	ko04530
Drug metabolism - other enzymes	16	717	0.002077023	ko00983
Biosynthesis of unsaturated fatty acids	6	142	0.002833766	ko01040
Cardiac muscle contraction	8	257	0.004027393	ko04260
Spliceosome	17	909	0.008783225	ko03040
Glutathione metabolism	8	306	0.01103588	ko00480
Protein processing in endoplasmic reticulum	16	897	0.01626663	ko04141
Hypertrophic cardiomyopathy (HCM)	8	334	0.01779255	ko05410
Biosynthesis of secondary metabolites	30	2047	0.01841002	ko01110
Allograft rejection	1	3	0.02910126	ko05330
Graft-versus-host disease	1	3	0.02910126	ko05332
PPAR signaling pathway	7	304	0.0309862	ko03320
Complement and coagulation cascades	4	124	0.03397758	ko04610
Arachidonic acid metabolism	5	183	0.03465662	ko00590
Glyoxylate and dicarboxylate metabolism	4	126	0.03571525	ko00630
Two-component system	3	78	0.04136638	ko02020
Terpenoid backbone biosynthesis	3	78	0.04136638	ko00900
Synthesis and degradation of ketone bodies	3	80	0.0440508	ko00072

1 The number of differentially expressed genes that belong to each KEGG pathway.

2 The number of expressed genes that belong to each KEGG pathway.

### Thiamethoxam-induced differential protein expression between susceptible and resistant *B. tabaci*

After challenged with thiamethoxam, 52 differentially expressed proteins (p-value≤0.05) were identified between susceptible (TH-S) and resistant (TH-2000) *B. tabaci*. Among them, 38 proteins were up-regulated (≥1.2∼Fold, p-value≤0.05) and 14 proteins were down-regulated (≤0.8∼Fold, p-value≤0.05) (Table S4). Following in-gel digestion by trypsin, peptides were identified by liquid chromatography-electrospray ionization/multistage mass spectrometry (LC-ESI-MS/MS; Table S4). Glutathione- S-transferase and glucosyl/glucuronosyl transferase, which are involved in xenobiotics detoxification, were up-regulated by 1.96 and 1.56-fold, respectively, in the resistant TH-2000 *B. tabaci* relative to the susceptible TH-S strain. Other up-regulated peptides in the resistant *B. tabaci* included UDP glucuronosyl transferase (1.73-fold), implicated in the inactivation and excretion of both endogenous and exogenous compounds [34]; luciferin regenerating enzyme (1.71-fold), playing an important role in the recycling of oxyluciferin into luciferin [35]; eukaryotic initiation factor (1.23-fold), associated with protein translation initiation and elongation processes [36]; glycyl-tRNA synthetase (1.38-fold), involved in RNA modification, RNA transportation, and amino acid-tRNA synthesis [37]; and ADP/ATP translocase proteins (1.49-fold), a group of enzymes catalyzing the exchange of ADP and ATP across the mitochondrial inner membrane [38]. In addition, proteins related to energy regulation, protein transportation and binding were also differentially expressed between the resistant and susceptible *B. tabaci* (Table S4).

**Figure 3:**
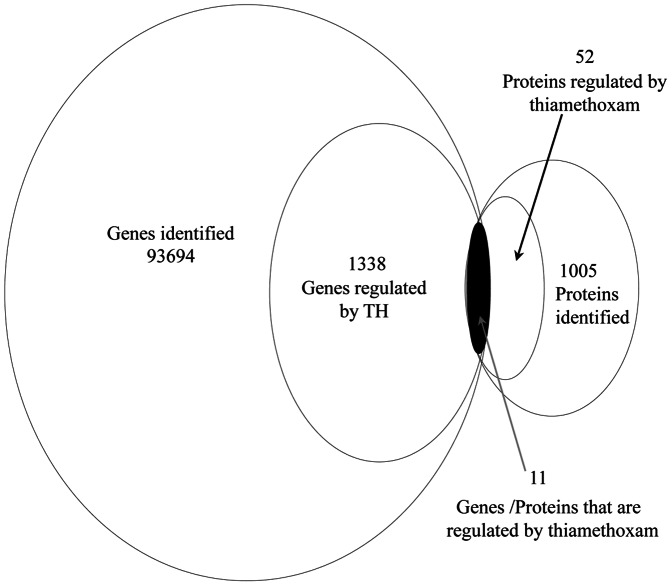
Correlation between the differently expressed proteins and genes. Scatter plots illustrates the distribution of differentially expressed proteins and related genes. The Pearson correlation coefficient between proteins and mRNA expression profiles is shown in the upper left corner of the plot.

To correlate protein with mRNA expression profiles, accession numbers from the proteomic dataset was extracted and compared to the annotated RNA-seq libraries. Differentially expressed peptides were compared to the nucleotide sequences using BLASTp. Differentially expressed peptides were compared to the nucleotide sequences using BLASTp as follows: (i) E-value is less than 1e-15, (ii) the number of mismatches is no more than 6%, and (iii) the alignment is at least 30 amino acids. Table S5 shows the directional correlation between mRNAs and proteins, and the correlation coefficient between the proteins and gene expression profiles was 0.6643 (Figure 3, Table S5).

### Gene ontology and pathway analysis

Among the 52 differentially expressed proteins, 28 were sub-categorized into 31 hierarchically-structured GO classes including 18 Biological Process, 8 Cellular Component, and 5 Molecular Function (Figure 2B). Specifically, “biological regulation” (9, 32.1%), “cellular process” (17, 60.7%), and “metabolic process” (14, 50%) were highly represented in “Biological Process”, whereas, “cell” (19, 67.9%), “cell part” (19, 67.9%), and “organelle” (12, 43%) were the most common categories in “Cellular Component”. In “Molecular Function”, the top three categories are “catalytic activity” (18, 64.3%), “binding” (10, 35.7%) and “transporter activity” (5, 17.9%) (Figure 2B, Table S6). Upon further investigation by GO enrichment analysis (p-value≤0.05), sodium/potassium-transporting ATPase subunit beta-2, mitochondrial import inner membrane translocase subunit TIM44, thioredoxin, serpin, cathepsin L, ruvB-like protein 1 isoform 1, transport protein sec23, mitochondrial ATP synthase coupling factor 6 precursor, 14-3-3 protein epsilon, peroxiredoxin-like protein, ribosomal protein S15Aa-isoform D, mitochondrial ATP synthase F chain, p270 proteins, GST, and cytochrome P450 *CYP3A* were significantly impacted by thiamethoxam treatment. To investigate which biological pathways were active when exposed to thiamethoxam, 35 differently expressed proteins were assigned to the reference pathways in KEGG. As a result, 14 pathways were enriched (p-value≤0.05), including “Metabolism of xenobiotics by cytochrome P450”, “Drug metabolism- cytochrome P450”, and “Drug metabolism-other enzymes”. Among them, “Metabolism of xenobiotics by cytochrome P450”, and “Drug metabolism- cytochrome P450” had the lowest p-value (Table 4). KEGG pathway analysis also revealed that the most-enriched peptides, including GSTs, UDP-glucuronosyltransferases, glucosyl/glucuronosyl transferases, and cytochrome P450s, were involved in xenobiotic metabolism (Table S7 and Table S8).

**Table 4:** Significantly enriched KEGG pathways in *B. tabaci* proteome.

Pathway	Protein		P-value	Pathway ID
	Dif Expressed^1^	Expressed^2^		
Metabolism of xenobiotics by cytochrome P450	6	16	3.10E-05	ko00980
Drug metabolism - cytochrome P450	6	16	3.10E-05	ko00982
Drug metabolism - other enzymes	6	20	0.000131541	ko00983
Steroid hormone biosynthesis	4	10	0.000597143	ko00140
Retinol metabolism	4	12	0.001318997	ko00830
Other types of O-glycan biosynthesis	3	6	0.001523666	ko00514
Ascorbate and aldarate metabolism	3	10	0.008066442	ko00053
Porphyrin and chlorophyll metabolism	3	11	0.01075072	ko00860
Aldosterone-regulated sodium reabsorption	2	4	0.01115195	ko04960
Pentose and glucuronate interconversions	3	15	0.02617927	ko00040
Bile secretion	2	6	0.02633193	ko04976
Carbohydrate digestion and absorption	2	6	0.02633193	ko04973
Protein digestion and absorption	2	7	0.03583105	ko04974
Proximal tubule bicarbonate reclamation	2	8	0.04643902	ko04964

1 The number of differentially expressed proteins that belong to each KEGG pathway.

2 The number of expressed proteins that belong to each KEGG pathway.

### qRT-PCR validation study

**Figure 4:**
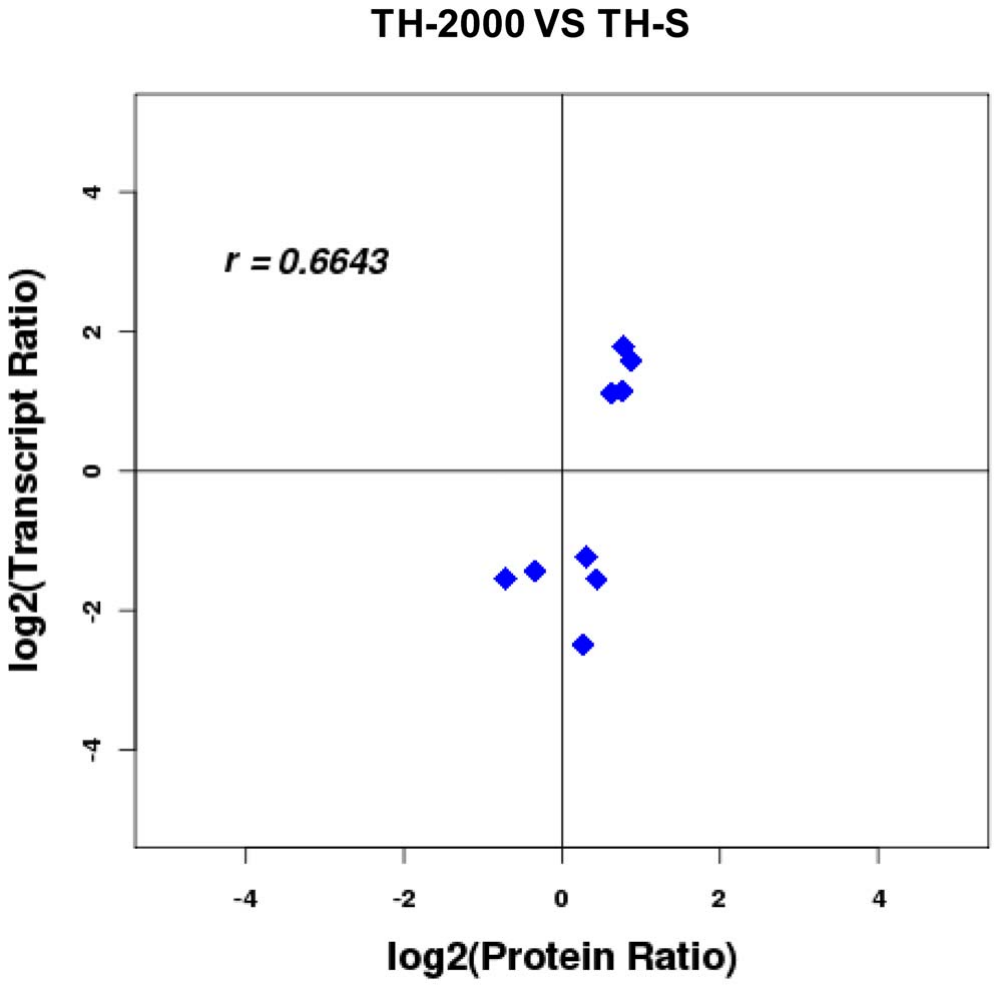
Quantitative real-time PCR analysis. The relative gene expression of selected target genes was normalized to a *BestKeeper* composed of endogenous reference genes *EF-1a* and *β-actin*. Standard errors were generated from the three biological replicates. Asterisks denote significant gene expression differences between resistant and susceptible *B. tabaci*, as determined by a paired t-tests (* p<0.05, ** p<0.01).

To validate results from transcriptomic and proteomic analyses, genes encoding differentially expressed proteins between thiamethoxam-resistant and -susceptible *B. tabaci* were subjected to the qRT-PCR analysis. Among the genes tested (41), 75% (31) were in agreement with the omics results (Table S9). Expression profiles of representative transcripts encoding UDP-glucuronosyltransferase 2B10-like isoform 1 (XP_002704642.1), glucosyl/glucuronosyl transferases (XP_969321.2), GST (ACH90394.1), and cytochrome P450 *CYP6CX1* (ACT78507.2), respectively, were shown in Figure 4. All four transcripts showed significantly higher expressions (p-value<0.05) in the resistant TH-2000 strain in comparison to the susceptible TH-S strain, in which *CYP6CX1* had the highest differential expression (∼11-fold).

### Enzymatic activity assay

Resistant *B. tabaci* strains (TH-R and TH-2000) were derived from a field-collected susceptible strain (TH-S), which had no previous exposure to insecticides. Both of these resistant strains have been exposed to thiamethoxam continuously for over 60 generations. Samples collected from TH-2000 were LC_80_ survivors, whereas TH-R were LC_50_ survivors. Theoretically, individuals from TH-2000 have a higher level of tolerance to thiamethoxam treatment, and this difference was reflected in the metabolic enzyme activity assays. For example, GST activity of TH-R strain was slightly higher than that of the susceptible TH-S strain, however, the difference was not substantial (one-way ANOVA, P<0.05; LSD test). In contrast, GST activity was significantly elevated in TH-2000 strain, which exhibited a 1.79-fold higher GST activity than TH-S when using CDNB and GSH as substrates (Table 5). The same trend was observed in P450 monooxygenase activities as well (Table 5). The PNOD activities of the two resistant TH-R and TH-2000 strains are 1.67 and 2.82-fold, respectively, greater than that of the susceptible TH-S strain. Furthermore, both GST and P450 activities between the two resistant strains (TH-R and TH-2000) were also significantly different (one-way ANOVA, P<0.05; LSD test) (Table 5).

**Table 5:** Metabolic enzyme activity among thiamethoxam resistance and susceptible *B. tabaci*.

Strain	GST activity (±SE)(OD340/min/mg)	Ratio		PNOD activity (±SE)(µmol/mg/30 min)	Ratio	
		TH-R/TH-S	TH-2000/TH-S		TH-R/TH-S	TH-2000/TH-S
TH-S	1.12 (±0.0769) B^1^	1.201	1.794	5.86 (±0.7636) C	1.672	2.816
TH-R	1.35 (±0.0799) B			9.79 (±2.1545) B		
TH-2000	2.02 (±0.1118) A			16.50 (±1.4669)A		

1 Means with different letters denote significant difference (P<0.05).

## Discussion

Insects are surrounded with an environment filled with toxic compounds, and constantly expose to xenobiotics through physical contact, ingestion, and inhalation. The repetitive application of synthetic insecticides in an artificially simplified (e.g., monoculture) agricultural ecosystem, insects develop an intricate detoxification system to maximize their survivorship by metabolizing insecticides and plant secondary compounds into less toxic substances and/or increasing their solubility to facilitate their sequestration [39]. Typically, the metabolic detoxification system in insects consists of three major groups of enzymes. The phase I detoxification enzymes, acting on a broad range of substrates directly to reduce their toxicity, are represented by the cytochrome P450 monooxygenases (P450s). The phase II enzymes, including glutathione S-transferases (GSTs), UDP-glucuronosyltransferases(UGTs), and carboxylesterases (COE), facilitate the excretion of hydrophobic toxic compounds by improving their hydrophilicity. The phase III enzymes are mainly composed of ATP binding cassette (ABC) and other transmembrane transporters that actively pump the conjugated xenobiotics out of the cell. A total of 196 genes encoding Phase I and II detoxification enzymes have been identified in *D. melanogaster* including 89 P450s, 39 GSTs, 35 COEs, and 33UGT [40], [41], [42]. In this study, the differentially expressed genes identified from transcriptome and proteome are predominantly Phase I and II detoxification enzymes, except one overexpressed Phase III enzyme, ABC sub-family G member 4-like isoform 1. Enzymatic activity assay results from this study and a previous report [26] clearly demonstrate the elevated metabolic enzyme responses toward thiamethoxam in *B. tabaci*.

### Phase I enzyme involved in thiamethoxam resistance

Cytochrome P450s (CYP, EC 1.14.14.1) are an extremely important metabolic system involved in catabolism of various classes of insecticides, and the CYP6 family has been implicated in insecticide resistance more than any other P450 families [43], [44]. Increased expression of *Cyp6g1* led to resistance to DDT, neonicotinoids, and insect growth regulators in *D. melanogaster* [4], [45]. Elevated expression of *CYP6D1*, *CYP6A*, and *CYP6D3* were associated with pyrethroid and neonicotinoids resistance in *M. domestica* [6], [46]. A brain-specific cytochrome P450, *CYP6BQ9*, was determined to be responsible for the deltamethrin resistance in *Tribolium castaneum* [47]. Two duplicated P450 genes, *CYP6P4* and *CYP6P9*, were over-expressed in pyrethroid resistant *Anopheles funestus* [48]. In *Anopheles gambiae*, *CYP6P3* was significantly over-expressed in field populations and this P450 was linked directly to the permethrin resistance [49].

In *B. tabaci*, increased detoxification by cytochrome P450s has been associated with neonicotinoid resistance. *CYP6cm1*, which was over expressed in several laboratory-selected *B. tabaci* strains, was considered as a legitimate molecular marker for screening imidacloprid resistance in the field [24]. The expression levels of *CYP6C* and *CYP9F*, respectively, were elevated 5–7 fold in imidacloprid resistant *B. tabaci* [50]. After more than 30 generations of selection, thiamethoxam resistance reached 66-fold in resistant *B. tabaci* [25], [26]. Synergism studies revealed that piperonyl butoxide, a cytochrome P450 inhibitor, had significant synergistic effects toward thiamethoxam, in which resistant ratio dropped to 3-fold in resistant *B. tabaci* [26]. In this study, combined data from omics analyses and enzymatic activity assays are in accordance with previous reports, strongly suggesting the involvement of P450 monooxygenases in thiamethoxam resistance in *B. tabaci*. Specifically, *CYP6CX1* exhibited significantly higher mRNA expression in a laboratory-selected thiamethoxam resistant strain. In addition, *CYP6CX1* has recently been implicated in *B. tabaci* resistance to fenvalerate, chlopyrifos, and avermectin in the field [51]. To elucidate its role in thiamethoxam resistance, molecular cloning and RNAi-based functional characterization are currently in progress.

### Phase II enzyme involved in thiamethoxam resistance

Similar to Phase I P450s, Phase II detoxification enzymes were also elevated in response to insecticide exposures. Elevated production of Phase II enzymes, GSTs (EC 2.5.1.18), has been documented as a mechanism of resistance to organochlorines, organophosphates (OP), and pyrethroids [52]. Over-expression of *GSTe2* led to resistance to both DDT and permethrin in *Aedes Aegypti* [53]. Elevated GST activity was also linked with acaricide resistance in *Tetranychus urticae* [54]. *MdGST-3* was over expressed in OP-resistant *M. domestica* [55]. In a lambda-cyhalothrin resistant *N. lugens*, the expression of *nlgst1-1* was highly elevated in comparison to a susceptible strain [56]. In the decamethrin resistant *Tenebrio molitor*, GST provided a passive protection mechanism by binding to this pyrethroid insecticide [57]. In this study, GSTs in a resistance TH-2000 strain was significantly over-expressed in comparison to the susceptible TH-S strain, and it is consistent with a previous study utilizing the same thiamethoxam resistant and susceptible *B. tabaci* strains [26]. This result is supported by the differentially expressed GSTs identified from both transcriptomic and proteomic analyses, indicating that GSTs play a central role in the detoxification of thiamethoxam in *B. tabaci*.

UDP-glucuronosyl transferases (EC 2.4.1.17; UGTs) are a major class of phase II drug metabolizing enzymes that play an important role in detoxifying a large number of xenobiotics through conjugation reactions [58]. Specially, the biological process predominantly involved UGTs is glucuronidation, in which a glucuronic acid moiety is added to a suite of endogenous and/or exogenous substrates to facilitate the removal of some of these compounds [59]. UGTs are highly expressed in liver and they are responsible for the metabolism of over 200 drugs in human body [58], [60]. Insect UGTs, on the other hand, are implicated in the detoxifications of insecticides [34]. UGTs activity was highly expressed in the larval and adult stages in *D. melanogaster*, and correlated with increased detoxification capability [61]. In *Bombyx mori*, expressed *BmUGT1* can conjugate a wide range of substrates including flavonoids, coumarins and other phenolic compounds, suggesting a role of UGTs in detoxification processes [62]. Besides detoxification, UGTs are also involved in olfaction, endobiotic modulation, UV protection, and sequestration [63]. In this study, UGT transcripts and proteins were increased 3.43 and 1.73-fold, respectively, in response to thiamethoxam exposure. Although there is no direct evidence linking UGTs with neonicotinoid resistance, the apparent over-expression of UGT at both transcription and translation levels in the resistant *B. tabaci* suggests the potential involvement of UGTs in thiamethoxam resistance.

Mitochondria is an important target site for toxic compounds and its involvement in apoptosis controls life and death decisions in the cell [64], [65]. Mitochondria cytochrome c oxidase (COX) (EC 1.9.3.1) is a complex metalloproteinase that provides a critical function in cellular respiration in both prokaryotes and eukaryotes [66]. In mammalian brain, reduced *COX* activity is an important factor for graded neuronal depolarization (or hyperexcitability) and neuronal death [67]. Elevated expression of *COX* was involved in the development of resistance to methotrexate in Chinese hamster ovary cells [68]. In *Blattella germanica*, *COX1* was over-expressed in a permethrin resistant strain [69]. Also, increased expression of *CO3* was tightly regulated in response to permethrin treatment in *A. Aegypti* [70]. In *Schistosoma mansoni*, *SCOX1* was substantially increased in a praziquantel resistant strain [71]. In this study, the *COX* is over-expressed in the TH-2000 strains, suggesting that cytochrome c oxidase may play a role in thiamethoxam resistant in whiteflies.

Enhanced production of carboxylesterases through gene amplification and/or up-regulation has been demonstrated in resistance to organophosphates, carbamates and pyrethroids in *M. persicae*, *Schizaphis graminum*, and *N. lugens* [72], [73], [74]. Although our transcriptomic and proteomic results did not identify differentially expressed carboxylesterases between resistant and susceptible *B. tabaci* showed specific activity of carboxylesterases was significantly higher in thiamethoxam resistance strain [26]. This is consistent with a previous study focusing on neonicotinoid cross-resistance in a *B. tabaci* B biotype in Israel [19], and indicates the potential involvement of carboxylesterases in thiamethoxam resistance. In a previous effort, a total of 72 and 52 up- and down-regulated transcripts were identified from the forward and reverse SSH libraries, respectively [27]. Similar to this study, differentially expressed genes between the thiamethoxam-resistant and -susceptible *B. tabaci* include, but not limit to, cell communication, response to abiotic stimulus, response to stress, lipid particle, nuclear envelope, cell proliferation, and nutrient reservoir activity. qRT-PCR analysis, however, validated 50% of the randomly selected transcripts showed significant differences. With the advent of Omics Era, transcriptomic and proteomic responses of *B. tabaci* to thiamethoxam were studied here using RNA-seq and iTRAQ, respectively. Ultra high-throughput omics analyses not only provided 10-fold more differentially expressed transcripts (1338 in comparison to 124), but also identified 52 differentially expressed proteins. Besides drastically improved sequencing output, qRT-PCR validation study indicates that coordinated omics approach (75%) is more accurate than SSH method (50%).

### Correlations between mRNA and protein expression

**Figure 5:**
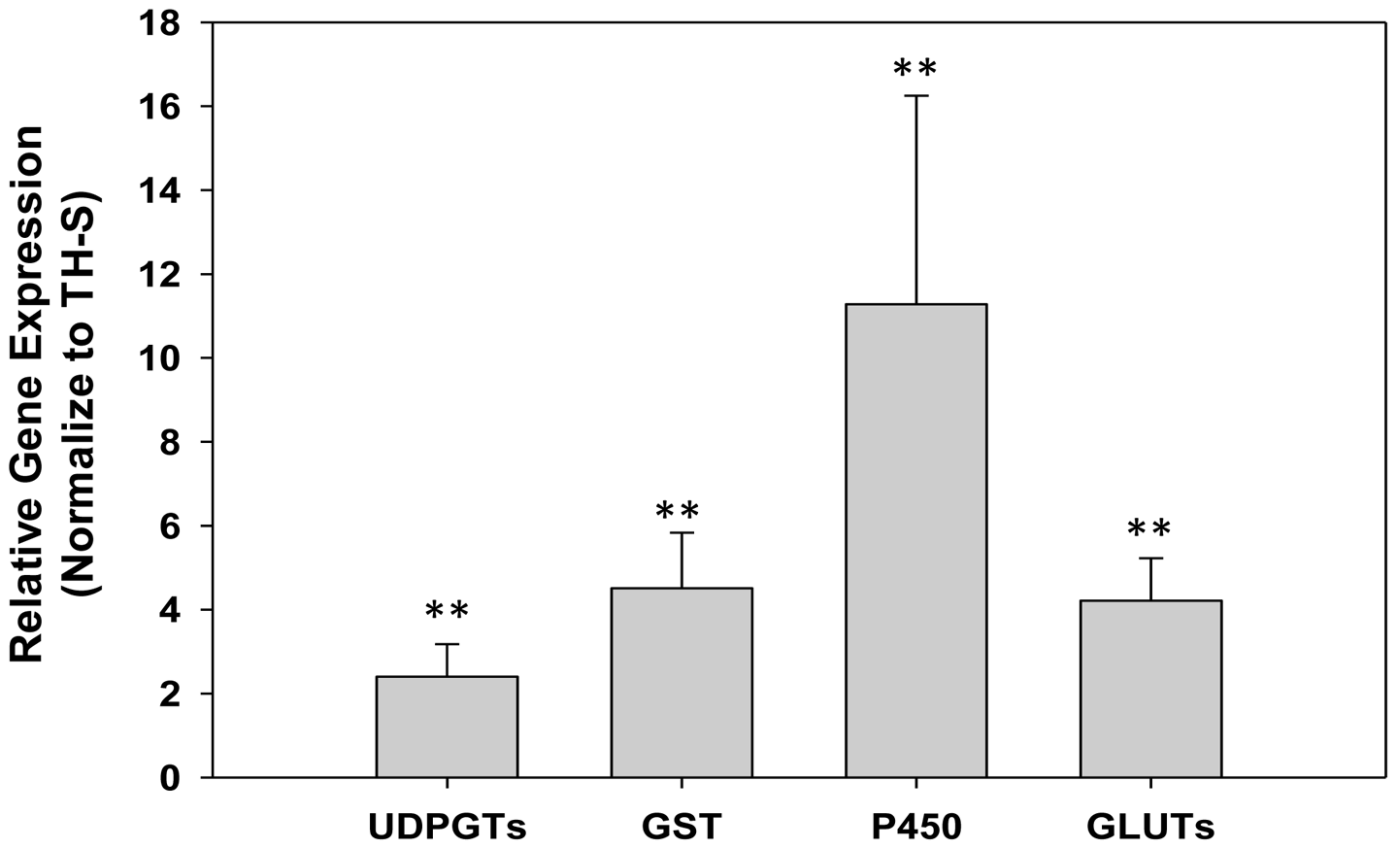
Venn diagram summarizing the proportion of proteins and genes significantly expressed between thiamethoxam resistance and susceptible *B. tabaci*. Differentially expressed transcripts and peptides are represented in respective circles. The overlapping region denotes specific transcripts with their corresponding peptides.

In this study, only a modest set of genes and proteins were concordantly differentially expressed (Figure 5). Expression profiles are highly dynamic, and mRNA expression and protein levels do not always correlate [75], [76]. Discrepancies in the directional changes between transcriptome and proteome are likely due to the single sampling time-point, as the temporal changes in transcript versus protein *in vivo* are poorly understood [77]. In addition, the regulatory mechanism of transcriptome and proteome are complex, and both turnover and mRNA stability are important factors contributing to translation efficiency [76], [77]. Furthermore, without a fully sequenced *B. tabaci* genome, difference between the expressed transcripts and the translated proteins will most likely be the norm rather than the exception.

### Summary

The exposure to xenobiotics typically induces a global transcriptional response that leads to the over expression of the detoxification machinery in insects. Our previous and current efforts using conventional SSH approach, coordinated omics analyses, and metabolic pathway analysis identified a core set of differentially expressed genes and proteins between the thiamethoxam-resistant and -susceptible *B. tabaci*. Specifically, the expression of a suite of Phase I and Phase II detoxification enzymes, including cytochrome P450s, UDP-glucuronosyltransferases, and glutathione S-transferases, were substantially elevated, suggesting a metabolic detoxification mechanism underlying the thiamethoxam resistance. Taken collectively, our results point the way to multiple avenues of “omics” approaches into the molecular understanding of insecticide resistance in an agriculturally important insect pest.

## Materials and Methods

### Ethics statement

*Bemisia tabaci* B biotype strains used in this study were initially collected in the field at Beijing in 2000, and have been maintained in a greenhouse at the Institute of Vegetables and Flowers, Chinese Academy of Agricultural Sciences. The species in the genus of Aleyrodidae are common agricultural pests and are not included in the “List of Protected Animals in China”. No specific permits were required for the described field studies.

### Sample preparation

Thiamethoxam susceptible (TH-S) and resistant (TH-R) *B. tabaci* strains were maintained on cabbage, *Brassica oleracea L.*, cv. Jingfeng 1 [25]. TH-S strain has been cultured without exposure to any insecticides [25]. In contrast, TH-R has been exposed to thiamethoxam for more than 60 generations, and exhibited >70-fold resistance to thiamethoxam in comparison to TH-S strain [26], [27]. In addition, a total of 10,000 TH-R adults were treated with 2,000 mg/L of thiamethoxam to eliminate the heterozygous individuals. After 48 hours, survivors were collected and designed as the TH-2000. This concentration (LC_80_) was determined based on a dose-response curve generated from an *in vivo* toxicity bioassay, in which 80% individuals from the resistance TH-R strain were killed by 2,000 mg/L of thiamethoxam insecticide. Approximately 1,500 TH-S and TH-2000 adults were collected, respectively, and snap-frozen immediately in liquid nitrogen and stored at −80°C.

### RNA-seq library preparation and sequencing

Total RNA was isolated from TH-S and TH-2000 whitefly samples, respectively, with Trizol (Invitrogen) and according to the manufacturer's protocol. The quantity and quality of RNA were determined with Nanodrop ND-1000. To remove residual DNA contamination, total RNA was treated with RNase-free DNase I (New England BioLabs). mRNA was purified from 6 ug of total RNA from each sample with Dynal oligo (dT) beads (Invitrogen), and then fragmented using RNA fragmentation kit (Ambion). The first cDNA strand was synthesized using random hexamer primers. The double-stranded cDNA fragments were processed by an end repair using T4 DNA polymerase, Klenow DNA polymerase, and T4 polynucleotide kinase (NEB) followed by a single Adenine base addition using Klenow 3′ to 5′ exo-polymerase, and concluded by ligation with Illumina's adaptor. The products were purified with QiaQuick PCR extraction kit (QiaGen) and enriched by PCR amplification. Finally, the library products were then subjected to sequencing analysis on the flow cell via Illumina HiSeq™ 2000.

### Annotation and *de novo* gene expression

Raw reads were transformed into clean reads by removing adaptor sequences, empty sequences (sequences with only adaptor but no reads), low-quality sequences (reads with unknown sequences [“N”]), and reads with a copy number of 1 (likely a sequencing error). All clean reads were then mapped to references sequences [32], [33] and only allowed no more than 1 nucleotide mismatch. Clean reads that map to multiple genes were filtered, and the remaining clean reads were designated as unambiguous reads. We, therefore, quantified transcript levels in reads per kilobase per million mapped reads (RPKM) [78]. For functional annotation, distinct sequences were BLAST against the NCBI NR database with a cut-off E-value of 10^−5^. In addition, Blast2GO (http://www.blast2go.org) was used to assign Gene Ontology terms (http://www.geneontology.org), while Kyoto Encyclopedia of Genes and Genomes (KEGG, http://www.genome.jp/kegg/ or http://www.kegg.jp/), a database resource that integrates genomic, chemical, and systemic functional information, was adopted to annotate molecular networks (pathways).

### Screening for differentially expressed genes

The mapped reads were assembled using a software package SOAPdenovo (short oligonucleotide alignment program) [79]. Then, the RPKM value for each transcript was measured in reads per kilobase of transcript sequence per million mapped reads. The significance of digital gene expression profiles were analyzed as described previously [80] with minor modifications. This algorithm, similar to GFOLD [81], denotes the number of unambiguous clean reads from gene A as x, as every gene's expression occupies only a small part of the library. P(x) yields to the Poisson distribution.
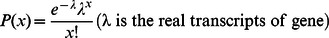
The fold change of each transcript was then calculated by the formula of log2 (TH-2000_RPKM/TH-S_RPKM). Where N1 and N2 indicate the total number of clean reads in TH-S and TH-2000, respectively, and x and y denote the mapped clean reads in each sample. FDR (False Discovery Rate) method was used to determine the threshold of p-value in differential gene expression tests. The probability that gene A expresses equally between two samples can be calculated with the following formula:
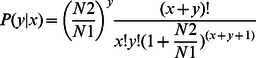
In this study, “FDR≤0.001 and the absolute value of log2Ratio≥1” was the threshold to evaluate the significance level of differentiated gene expression.

### Protein quantification and database search using iTRAQ labeling

1 mg of whiteflies (approximately 100 individuals) were dissected in lysis buffer (7M urea, 2M thiourea, 4% CHAPS, 40 mM Tris-HCl, pH 8.5), and 1 mM PMSF (phenylmethanesulfonyl fluoride) and 2 mM EDTA (ethylene diamine tetraacetic acid) were added later, respectively. After 5 min, 10 mM DTT was added to the lysis solution, and then it was centrifuged at 4°C, 30,000×*g* for 15 min. The supernatant was collected, and the concentration of total proteins was determined using a 2D Quantification Kit (GE Healthcare). For quality check, 30 µg of total protein from each sample was subjected to the SDS-PAGE analysis. After that, 100 µl protein from each sample was digested with trypsin gold (Promega) (protein∶ trypsin = 30∶1) at 37°C for 16 h, and resultant peptides were dried by vacuum centrifugation. The peptides were reconstituted in 0.5 M TEAB and processed according to the manufacturer's protocol for 8-plex iTRAQ (Applied Biosystems, Inc). Samples (100 mg total protein/sample) from TH-S and TH-2000 strains were labeled with 113 and 114 iTRAQ tags, respectively. Then pooled mixtures of iTRAQ-labeled peptides were fractionated by SCX chromatography (Phenomenex, Inc, USA) using a Shimadzu LC-20AB HPLC Pump system. Collected fractions were pooled into 10 final fractions and analyzed by nano LC-MS/MS analysis after desalting by Strata XC18 column (Phenomenex) and vacuum-dried. Nano LC-MS/MS analysis on each of these fractions was performed using a LTQ-Orbitrap Velos mass spectrometer (Thermo Fisher Scientific Inc. Rockford, IL., USA) equipped with nanoelectrospray ionization.

Peptides were identified by searching against a combined database containing *B. tabaci* B and Q biotypes (reference transcriptomes included both 454 and illumina sequencing data) [32], [33] using a MS/MS data interpretation algorithm within Mascot. Searches were restricted to whitefly database with carbamidomethyl cysteine as a fixed modification, and oxidized methionine as a variable modification. A peptide mass tolerance of 3 ppm and fragment mass tolerance of ±0.05 Da with one missed cleavage site of trypsin were allowed. When the Mascot software was used to search the database, 1005 proteins were identified with a false discovery rate (FDR) of less than 1%. Differential expression ratios for proteins were obtained from Mascot software (http://www.matrixscience.com), which calculates protein ratios using only ratios from the spectra that are distinct for each protein and excluding the shared peptides of protein isoforms. To calculate differential expression ratios, all identified spectra from a protein were used to obtain an average protein ratio relative to the control label (i.e. fold change). Student t-test was used to analyze the differential expression of proteins between resistant and susceptible *B. tabaci*. The p-value was calculated using the confidence intervals from the error factor generated in Mascot as
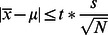
where, N is the number of peptide ratios, s is the standard deviation, and x represents the mean of peptide ratios. In this study, we used P≤0.05 and the fold change >1.2-fold or <0.8-fold as the thresholds to judge the significance level of differentiated protein expression.

### Functional and pathway enrichment analyses

Functional annotation of transcripts and proteins identified in *B. tabaci* was carried out using Blast2GO, an integrated GO annotation and data mining tool that assigns gene ontology through BLAST searches against nucleotide and/or protein databases [82]. GO enrichment analysis provides all GO terms that significantly enriched in differentially expressed genes/proteins in comparison to susceptible whiteflies. This method firstly maps all differentially expressed genes/proteins to GO terms in the database (http://www.geneontology.org/), calculating gene numbers for every term, then using hypergeometric test to find significantly enriched GO terms in differentially expressed genes/proteins comparing to *B. tabaci* transcriptome/proteome background. The calculating formula is
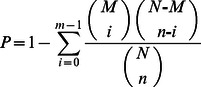
Where N is the number of all genes/or proteins with GO annotation; n is the number of differentially expressed genes/or proteins in N; M is the number of all genes/or proteins that are annotated to the certain GO terms; m is the number of differentially expressed genes/or proteins in M. The calculated p value was subjected to a Bonferroni Correction, taking a corrected p-value of 0.05 as a threshold. GO terms fulfilling this condition were defined as significantly enriched GO terms in differentially expressed genes/or proteins.

All identified transcripts and proteins were mapped to pathway in the Kyoto Encyclopedia of Genes and Genomes (KEGG) database. Significantly enriched metabolic pathways or signal transduction pathways in differentially expression genes and proteins were identified using the same calculating formula as in GO analysis. Here N is the number of all genes/or proteins that with KEGG annotation, n is the number of differentially expressed genes/or proteins in N, M is the number of all genes/or proteins annotated to specific pathways, and m is number of differentially expressed genes/or proteins in M.

### Correlation between protein and mRNA expression

To assess the correlation between transcriptomic and proteomic platforms, we first set cutoff values to select subsets of genes and proteins with distinctive expression signals. All the protein sequences identified by iTRAQ were analyzed and converted into a searchable database. For each protein, we queried the RNA-seq data from the same expression pattern of a matching transcript (P-value<0.05). The significance level of the overlap between detected proteins and transcripts was determined using Pearson's Chi-squared test with Yates' continuity correction.

### Quantitative real time PCR (qRT-PCR) analysis

Total RNA was extracted from TH-2000 and TH-S adults, respectively, using Trizol (Invitrogen) following the manufacturer's protocols. The total RNA obtained was resuspended in nuclease-free water and the concentration was measured using Nanodrop (Thermo Scientific Nanodrop 2000). cDNA was synthesized using the SYBR PrimeScript reverse transcription-PCR (RT-PCR) kit (Takara). qRT-PCRs were carried out on an ABI 7500 real-time PCR system (Applied Biosystems) with SYBR Green Real-time PCR Master Mix (TaKaRa) following a cycling regime of 95°C for 3 min, 40 cycles of 95°C for 30 s, 60°C for 30 s, and 72°C for 35 s. The primers used for real-time PCR are listed in Table S10. Three biological replicates for each sample were used for qRT-PCR analysis. The relative gene expression of selected target genes was normalized to the *BestKeeper* [83], a composite internal reference gene composed of endogenous *EF-1a* and *β-actin* to eliminate sample-to-sample variations. The relative expression levels were calculated using the 2^−ΔΔCt^ method [84].

### Enzymatic activity assays

GST activity was measured using the 1-chloro-2,4-dinitrobenzene (CDNB) and reduced GSH as substrates according to Feng [29]. The total reaction volume per well of a 96-well microtiter plate total volume was 300 µL, consisting of 100 µL crude enzyme,100 µL 1-chloro-2,4-dinitrobenzene (CDNB) (1.2 mM), and 100 µL glutathione reduced (GSH) (12 mM). Change in absorbance was monitored continuously at 340 nm for 10 min at 25°C using a thermomax kinetic microplate reader (Molecular Devices). Cytochrome-P450-dependent monooxygenase activity was determined using *p*-nitroanisole (PNA) as substrates with minor modifications [29]. Incubation mixture contained 375 µL PNA (2 µM), 37.5 µL NADPH (Nicotinamide adenine dinucleotide phosphate) (9.6 mM), and 337.5 µL crude enzyme. The mixture was incubated for 30 min at 34°C in one air atmosphere. The reaction volume per well of a 96-well microtiter plate was 200 µL of incubation mixture. The P450 monooxygenase activity is determined spectrophotometrically by monitoring the accumulation of metabolic products according to a p-nitrophenol standard curve at OD405.

## Supporting Information

Dataset S1 **Peptide spectra in** ***B. tabaci*** **proteome.**
(XLSX)

Table S1 **Gene ontology analysis of** ***B. tabaci*** **transcriptome.**
(XLSX)

Table S2 **Differentially expressed genes within drug metabolism pathway in response to thiamethoxam exposure.**
(XLSX)

Table S3 **Differentially expressed genes within metabolism pathways in response to thiamethoxam exposure.**
(XLSX)

Table S4 **Differentially expressed peptides identified by LC-ESI-MS/MS.**
(XLSX)

Table S5 **Differentially expressed peptides and their corresponding mRNAs in response to thiamethoxam exposure.**
(DOCX)

Table S6 **Gene ontology analysis of** ***B. tabaci*** **proteome.**
(XLSX)

Table S7 **Differentially expressed peptides within drug metabolism pathway in response to thiamethoxam exposure.**
(XLSX)

Table S8 **Differentially expressed peptides within metabolism pathways in response to thiamethoxam exposure.**
(XLSX)

Table S9 **qRT-PCR verified differentially expressed genes.**
(XLSX)

Table S10 **Primers used for qRT-PCR analyses.**
(XLSX)
