# Supplementary material for: Transcriptomic and Proteomic Responses of Sweetpotato Whitefly, Bemisia tabaci, to Thiamethoxam
Source: PLoS One. 2013 May 9;8(5):e61820. doi: 10.1371/journal.pone.0061820 (PMC3650016; doi:10.1371/journal.pone.0061820)
Supplement: Table S5 — Differentially expressed peptides and their corresponding mRNAs in response to thiamethoxam exposure. (DOCX) [file pone.0061820.s006.docx]

**Table S5. Differentially expressed peptides and their corresponding mRNAs in response to thiamethoxam exposure.**

| **Corresponding mRNA** | | | **Protein** | |
| --- | --- | --- | --- | --- |
| **Gene ID^a^** | **Description** | **FC^b^** | **Description** | **FC^b^** |
| 1 | Glucosyl/glucuronosyl transferases | 2.15 | Glucosyl/glucuronosyl transferases | 1.56 |
| 2 | Luciferin-regenerating enzyme | 2.2 | Luciferin regenerating enzyme | 1.71 |
| 3 | Glutathione-S-transferase | 2.99 | Glutathione-S-transferase | 1.84 |
| 4 | MGC82850 protein | 2.24 | MGC82850 protein | 1.54 |
| 5 | UDP glucuronosyltransferase 2B10 | 3.43 | UDP glucuronosyltransferase 2B10-like isoform 1 | 1.73 |
| 6 | Glutathione S-transferase | 1.27 | Glutathione S-transferase | 1.96 |
| 7 | Proteasome subunit alpha type 1 | 0.64 | Proteasome subunit alpha type 1 | 1.29 |
| 8 | RuvB-like protein 1 isoform 1 | 1.11 | RuvB-like protein 1 isoform 1 | 1.35 |
| 9 | Mitochondrial thioredoxin 2 | 1.28 | Thioredoxin, mitochondrial | 1.29 |
| 10 | Similar to serpin | 0.77 | Similar to serpin | 1.22 |
| 11 | Mitochondrial ATP synthase Coupling factor 6 precursor | 1.46 | Mitochondrial ATP synthase coupling factor 6 precursor | 1.49 |
| 12 | Similar to protein transport protein sec23 | 0.84 | Similar to protein transport protein sec23 | 1.3 |
| 13 | Similar to ENSANGP00000024769 | 0.73 | Quaking related 54B CG4816-PB, isoform B | 1.2 |
| 14 | Ribosomal protein S15Aa, isoform D | 1.08 | Ribosomal protein S15Aa, isoform D | 1.26 |

**Table 8. Count.**

| **Protein** | |  | **Corresponding mRNA** | |
| --- | --- | --- | --- | --- |
| **Accession NO.*^a^*** | **Description** | **FC *^b^*** | **Description** | **FC *^b^*** |
|  |  |  |  |  |
| 15 | Developmentally regulated GTP-binding protein 2 | 0.94 | GTP-binding protein 128up | -0.8 |
| 16 | Glycyl-tRNA synthetase | 1.7 | Glycyl-tRNA synthetase | 1.38 |
| 17 | Cathepsin L precursor | 0.71 | Cathepsin L | 1.27 |
| 18 | Eukaryotic translation initiation factor 3 subunit | 0.87 | Eukaryotic translation initiation factor 3 subunit | 1.23 |

Significantly differently expressed proteins between thiamethoxam resistance and susceptible *B. tabaci* identified by iTRAQ (p-value<0.05, FDR adjust), and further significantly differently expressed mRNA corresponding to the proteins identified by RNA-seq (FDR<0.001) were all shown in this table.

Negative value indicates down-regulated. Black value indicates no significance was detected. mRNAs were annotated using Blastx Descriptor Annotator with default values (cutoff evalue≤1.0E-5).

*“a”:* Gene ID contains Isotig/ Sington ID and sequence information available upon request.

“*b”:* Fold change, gene or protein expression level.
